# Accidental Intralenticular Injection of Ozurdex® for Branch Retinal Vein Occlusion: Intact Posterior Capsule and Resolution of Macular Edema

**DOI:** 10.1155/2019/8630504

**Published:** 2019-01-23

**Authors:** Ali Kurt, Ali Hakan Durukan, Murat Küçükevcilioğlu

**Affiliations:** ^1^Ahi Evran University Faculty of Medicine, Department of Ophthalmology, Turkey; ^2^Health Sciences University Faculty of Medicine, Department of Ophthalmology, Turkey; ^3^Gülhane Education and Research Hospital, Department of Ophthalmology, Turkey

## Abstract

**Purpose:**

We present a case of accidental intralenticular injection of Ozurdex implant in a patient with macular edema secondary to branch retinal vein occlusion.

**Method:**

A case report.

**Results:**

Intravitreal dexamethasone implant injection had been performed for macular edema due to left superior temporal vein branch occlusion to the left eye of a 78-year-old male patient. The slit-lamp examination 85 days later revealed that the dexamethasone implant was intralenticular. The best-corrected visual acuity (BCVA) was 0.16 on the Snellen chart. Cataract surgery was decided on for the cataract as there was no anterior chamber inflammation, the intraocular pressure (IOP) was normal, and the macular edema had resolved. Uneventful phacoemulsification within the bag intraocular lens placement was performed.

**Conclusions:**

Accidental intralenticular Ozurdex injection is an extremely rare complication. The surgeon must decide whether to continue to observe or intervene immediately when such a complication is encountered. Cataract surgery can be planned if the macular edema has resolved and a cataract has developed. It is important to evaluate the posterior capsule with ultrasound biomicroscopy and Scheimpflug imaging before the cataract surgery to ensure a safe surgical procedure.

## 1. Introduction

Retinal vein occlusion (RVO) is only surpassed by diabetic retinal disease as a retinal vascular disorder. The two main types are branch retinal vein occlusion (BRVO) and central retinal vein occlusion (CRVO). The main cause of decreased visual acuity with BRVO is the cystoid macular edema (CME) that develops in 30% of the cases [1]. Dexamethasone intravitreal implant (Ozurdex®; Allergan, Inc., Irvine, Calif., USA) has been approved by the Food and Drug Administration in the USA to treat the macular edema seen with RVO, in addition to central diabetic macular edema and noninfectious posterior uveitis [2].

Increased intraocular pressure (IOP) and the onset or progression of cataracts are the most common adverse ocular reactions with Ozurdex use [3]. However, there have also been increasing reports of accidental injection of Ozurdex into the crystalline lens [3–13]. Some authors feel expectant waiting is better [3, 4, 7, 10, 11] while others suggest immediate phacoemulsification (PE) for the cataract [5, 6, 13].

We present a case of accidental intralenticular Ozurdex implant injection where the macular edema recovered and was followed by uneventful PE.

### 1.1. Case Presentation

**Figure 1:**
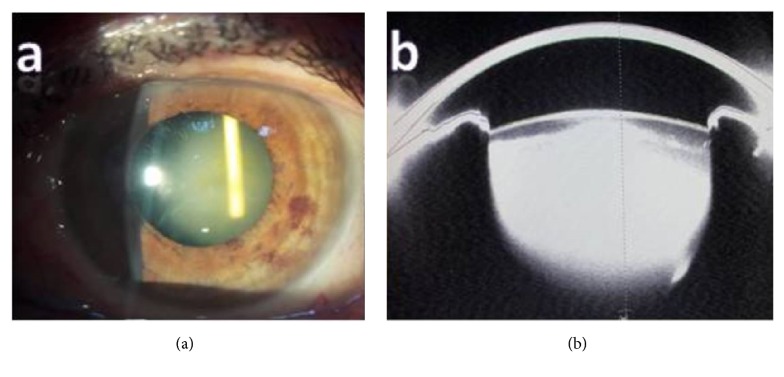
(a) Slit lamp photograph showing the intralenticular Ozurdex implant. (b) Scheimpflug image showing the intralenticular Ozurdex implant together with the intact posterior capsule.

**Figure 2:**
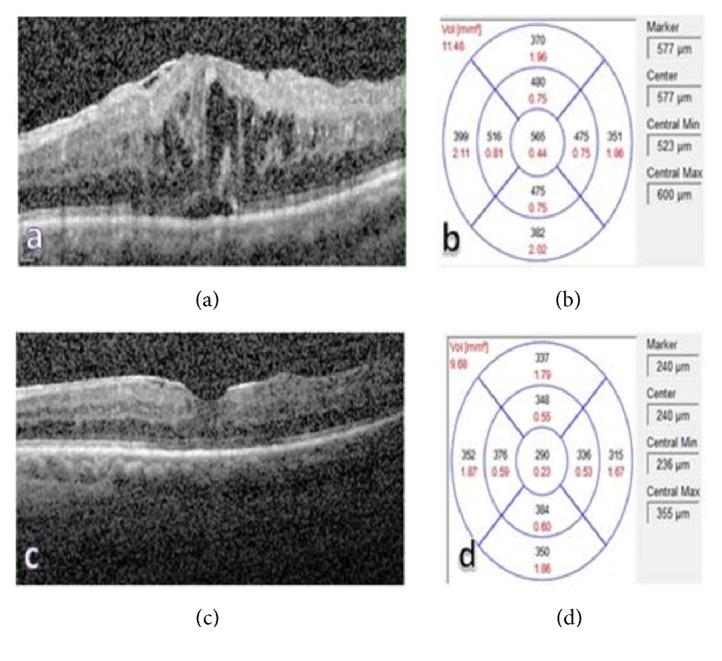
(a)-(b) Optical Coherence Tomography image showing the macular edema before Ozurdex injection (CMT: 565 *μ*m). (c)-(d) Optical Coherence Tomography images 85 days after the following Ozurdex administration showing resolution of the macular edema (CMT: 290 *μ*m).

A hypertensive and diabetic 78-year-old male presented with decreased left visual acuity. There was a history of intravitreal Ozurdex implantation approximately 85 days ago for upper temporal RVO and CME. The best-corrected visual acuity (BCVA) was 0.16 on the Snellen chart and the IOP was 16 mmHg. Slit lamp examination revealed a Grade II nuclear cataract without anterior chamber inflammation and there was an intralenticular dexamethasone implant in the upper part of the lens (Figure 1(a)). Fundus examination revealed findings secondary to upper temporal RVO. Scheimpflug photograph of the left eye showed the intralenticular Ozurdex implant with an intact posterior capsule (Figure 1(b)). Comparison of OCT images of the macular edema prior to Ozurdex injection (central macular thickness (CMT): 565 *μ*m) (Figures 2(a) and 2(b)) and 85 days afterwards (CMT: 290 *μ*m) demonstrated resolution of the edema (Figures 2(c) and 2(d)).

### 1.2. Surgical Procedure Outline

**Figure 3:**
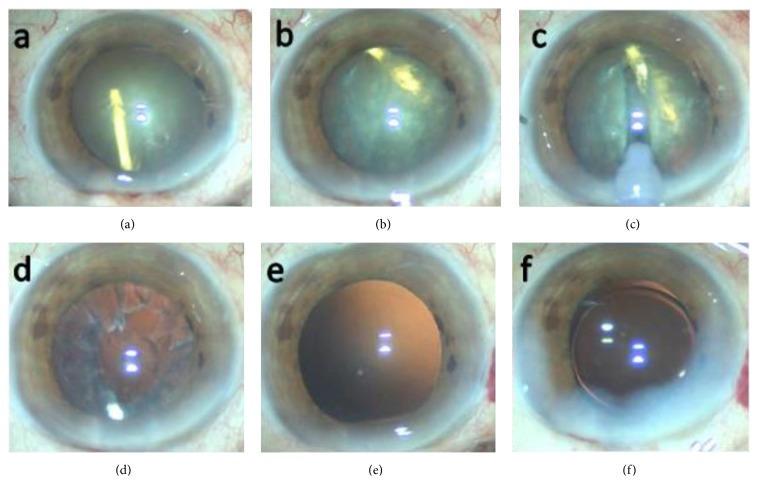
(a) Completed anterior continuous curvilinear capsulorrhexis, (b) image of intralenticular Ozurdex rotated with nucleus, (c) creating a groove with phaco probe, (d) cortical remnants, (e) image of intact posterior capsule, and (f) intraocular lens implantation.

Anterior continuous curvilinear capsulorrhexis was performed using viscoelastic material protection (Figure 3(a)). The nucleus was rotated following gentle hydrodelineation and hydrodissection. The intralenticular Ozurdex implant rotated together with the nucleus (Figure 3(b)). After a groove was created with the phaco probe (Figure 3(c)), the nucleus, and Ozurdex implant were easily removed. The cortical remnants (Figure 3(d)) were removed with irrigation-aspiration. We observed that the posterior capsule was intact (Figure 3(e)). A hydrophopic acrylic three-piece IOL (Sensar AR40e; Abbott Medical Optics, Santa Ana, California, USA) was then placed inside the capsule using 1% sodium hyaluronate (Figure 3(f)).

## 2. Discussion

Ozurdex (Allergan Inc., Irvine, CA, USA) is a rod-shaped biodegradable dexamethasone (0.7 mg) implant that is 6 mm in length and 0.46 mm in diameter. It is injected into the vitreous cavity through a 22-gauge needle. The implant is injected into the mid-vitreous, 3.5 mm to 4 mm posterior to the limbus. The muzzle velocity has been calculated as 0.8 m/s [14]. There have been many case reports of accidental Ozurdex injection in the crystalline lens in recent years [3–13]. The contributing factors are thought to be lack of experience or inappropriate technique on the surgeon's part and uncontrolled head movement during the procedure by the patient [3].

Accelerated cataract development has been reported in some intralenticular Ozurdex implant administration cases [4–6], while other cases had increased IOP [8] or both these complications together [9, 10]. Poornachandra et al. [11] and Clemente-Tomás et al. [3] have reported no acceleration of cataract progression. Many authors [3, 4, 7, 10, 11] have found a gradual resolution of the macular edema with an intralenticular implant but Baskan et al. [12] found decreasing vision and no edema improvement. Coca-Robinot et al. [9] have observed very little effect of the intralenticular implant on the macular edema.

An intralenticular implant can be monitored in several different ways. The wait-and-see approach and later cataract surgery are used by most authors [3, 4, 7, 10, 11]. In contrast, some authors [5, 6, 13] suggest early removal of the implanted Ozurdex device with phacoemulsification, followed by repositioning within the vitreous, but this approach can have several disadvantages. First of all, the potential for fibrosis development at the posterior capsule tear is eliminated. It is also possible for the increased posterior capsule stress during phacoemulsification to enlarge the tear. The implant repositioning in the vitreus cavity can be difficult because of vitreus resistance. The implant can also become fragmented during repositioning in the vitreus cavity with altered drug absorption and increased glaucoma risk [15]. Another risk of this approach is the migration of the reinserted implant to the anterior chamber through the posterior capsule defect, resulting in corneal decompensation [2].

We presented a case of intralenticular Ozurdex implantation where the macular edema had resolved and a cataract had accelerated developed 85 days later without an increase in IOP, necessitating phacoemulsification. We believe the resolution of the macular edema in our case was due to the release of the drug in small amounts from the part of the implant that contacted the vitreus outside the capsule and attributed the uncomplicated phacoemulsification surgery and in the bag IOL implantation success to the closure of the posterior capsule with fibrosis at the implant entry point.

In conclusion, accidental intralenticular injection of Ozurdex is an extremely rare complication. However, the surgeon must decide on whether to wait and see or intervene immediately when confronted with such a case. The various factors that need to be taken into account are decreased visual acuity (due to cataract progression or the implant obstructing the visual axis), increased intraocular pressure, and the state of the macula. Cataract surgery may be necessary when the macular edema is resolved and a cataract has developed. We believe using ultrasound biomicroscopy and Scheimpflug imaging to evaluate the posterior capsule before cataract surgery if possible will make it easier to foresee the potential complications that may develop during phacoemulsification.
